# Binocular Microscope

**Published:** 1853-03

**Authors:** 


From the New Orleans Med. and Surg. Journal.

*Binocular Microscope.*

(From the Transactions of the Phys. Med. Society of New Orleans.)—At a meeting of the Physico-Medical Society, on Saturday evening, 2d October, Prof. J. L. Riddell called the attention of the Society to an instrument of his own invention and manufacture, which promises to be of incalculable advantage in microscopic researches, especially in the prosecution of microscopic anatomy and physiology.

He remarked, that he last year contrived, and had lately constructed and used, a combination of glass prisms, to render both eyes serviceable in microscopic observation. The plan is essentially as follows:

Behind the objective, and as near thereto as practicable, the light is equally divided, and bent at right angles and made to travel in opposite directions, by means of two rectangular prisms, which are in contact by their edges, that are somewhat ground away. The reflected rays are received at a proper distance for binocular vision upon two other rectangular prisms, and again bent at right angles, being thus either completely inverted, for an inverted microscope, or restored to their original direction. These outer prisms may be cemented to the inner, by means of Canada balsam, or left free to admit of adjustment to suit different observers. Prisms of other form, with due arrangement, may be substituted.

This method proves, according to Prof. Riddell's testimony, eqally applicable to every grade of good lenses, from Spencer's best sixteenth to a common three inch magnifier, with or without oculars or erecting eye-pieces, and with great enhancement of penetrating and defining power. It gives the observer perfectly correct views in length, breadth and depth, whatever power he may employ; objects are seen holding their true relative positions, and wearing their real shapes. In looking at solid bodies, however, depressions sometimes appear as elevations, and vice versa, forming a curious illusion; for instance, a metal spherule may appear like a glass ball silvered on the under side, and the margin of a wafer may seem to ascend from the wafer into the air.

With this instrument the microscopic dissecting knife can be exactly guided. The watchmaker and artist can work under the binocular eye-glass with certainty and satisfaction. In looking at microscopic animal tissues, the single eye may perhaps behold a confused amorphous, or nebulous mass, which the pair of eyes instantly shape into delicate superimposed membranes, with intervening spaces, the thickness of which can be correctly estimated. Blood corpuscles, usually seen as flat disks, loom out as oblate spheroides. Prof. R. asserted, in short, that the whole microscopic world could thus be exhibited in a new light, acquiring a ten-fold greater interest, displaying in every phase a perfection of beauty and symmetry indescribfible.

